# A fully human anti-CD47 blocking antibody with therapeutic potential for cancer

**DOI:** 10.18632/oncotarget.13349

**Published:** 2016-11-15

**Authors:** Dadi Zeng, Qiang Sun, Ang Chen, Jiangfeng Fan, Xiaopeng Yang, Lei Xu, Peng Du, Weiyi Qiu, Weicai Zhang, Shuang Wang, Zhiwei Sun

**Affiliations:** ^1^ Beijing Institute of Biotechnology, Fengtai District, Beijing 100071, China

**Keywords:** CD47, anti-CD47 antibody, phagocytosis, cancer therapy, cell-in-cell

## Abstract

CD47/SIRPα interaction serves as an immune checkpoint for macrophage-mediated phagocytosis. Mouse anti-CD47 blocking antibodies had demonstrated potent efficacy in the treatment of both leukemic and solid tumors in preclinical experimentations, and therefore had moved forward rapidly into clinical trials. However, a fully human blocking antibody, which meets clinical purpose better, has not been reported for CD47 up to date. In this study, we reported the isolation of a fully human anti-CD47 blocking antibody, ZF1, from a phage display library. ZF1 displayed high specificity and affinity for CD47 protein, which were comparable to those for humanized anti-CD47 blocking antibody B6H12. Importantly, ZF1 treatment could induce robust, or even stronger than B6H12, phagocytosis of leukemic cancer cells by macrophage *in vitro*, and protect BALB/c nude mice from cancer killing by engrafted leukemic cells (CCRF and U937) to a similar extent as B6H12 did. Thus, these data provide primary early pre-clinical support for the development of ZF1 as a fully human blocking antibody to treat human leukemia by targeting CD47 molecule.

## INTRODUCTION

CD47 is one of the immunoglobulin superfamily protein members, harboring an IgV-like extracellular domain, five transmembrane motifs and a short intracellular domain [1, 2]. Physiologically, CD47 is a broadly expressed antigen, present on many different cell types in almost all tissues [2]. CD47 is involved in a number of cellular processes, including proliferation [3–5], apoptosis [6], adhesion, migration [7, 8] and also plays important roles in immune regulation, homeostasis and nervous systems [9–13] via binding with SIRPα [14, 15] TSP1 [16, 17]and integrin [18]. Pathologically, CD47 is highly expressed on many kinds of hematopoietic tumors, including acute myeloid leukemia (AML), chronic myeloid leukemia(CML), acute lymphoblastic leukemia (ALL), non-Hodgkin's lymphoma (NHL), multiple myeloma (MM) [19–22], and solid tumors from ovarian, breast, colon, and the like [23–25]. Studies have shown that high expression of CD47 correlates with a poor clinical prognosis and adverse molecular features in multiple cancer types [20–25].

CD47 enabled the cancer cell to evade immune-surveillance and attack by sending “don't eat me” signal to phagocytic cells, such as macrophages and dendritic cells (DCs), via binding SIRPα, which prevents phagocytosis and T cell activation [10, 11, 26]. Blocking CD47/SIRPα interaction could induce phagocytosis [19, 25, 27] and enable DCs to cross-present tumor antigens through MHC class I molecules, which further activates CD8^+^ T cells that are specific for tumor antigens [28, 29]. Additionally, studies have shown that ligation of CD47 could induce apoptosis of cancer cells [6, 30, 31] and modulate tumor microenvironment [8, 32].

Multiple strategies had been developed for cancer therapy by targeting CD47/SIRPα interaction. For example, the binding domain of human SIRPα was modified to produce high-affinity variants, which demonstrated anti-cancer effects either as single agents or when combined with rituximab [27]. Similarly, a CD47 variant was also developed for cancer immunotherapy [33]. Besides, anti-CD47 blocking antibodies represent one of the most successful way to achieve desirable anti-cancer therapeutic effects [11, 19–21, 25, 29, 34, 35]. Currently, several clinical trials for anti-CD47 monoclonal antibodies and other blocking agents are under way (NCT02216409, NCT02678338, NCT02367196, NCT02641002 and NCT02663518) (https://clinicaltrials.gov/). Nevertheless, the antibodies used are either mouse-origin or humanized [34], which might be disadvantageous for residual mouse amino acid sequence. To our knowledge, there is still no human anti-CD47 antibody reported. Here in this work, we developed a fully human anti-CD47 antibody with therapeutic potential by phage display library technique. The antibody, named ZF1, can block the interaction of CD47 and SIRPα, enhancing phagocytosis of leukemia cells by macrophages. Significantly, ZF1 showed remarkable anti-leukemia effect *in vivo*, which supports ZF1 as a promising human antibody for cancer therapy.

## RESULTS

### Phage display library screening for human anti-CD47 antibody

**Figure 1:**
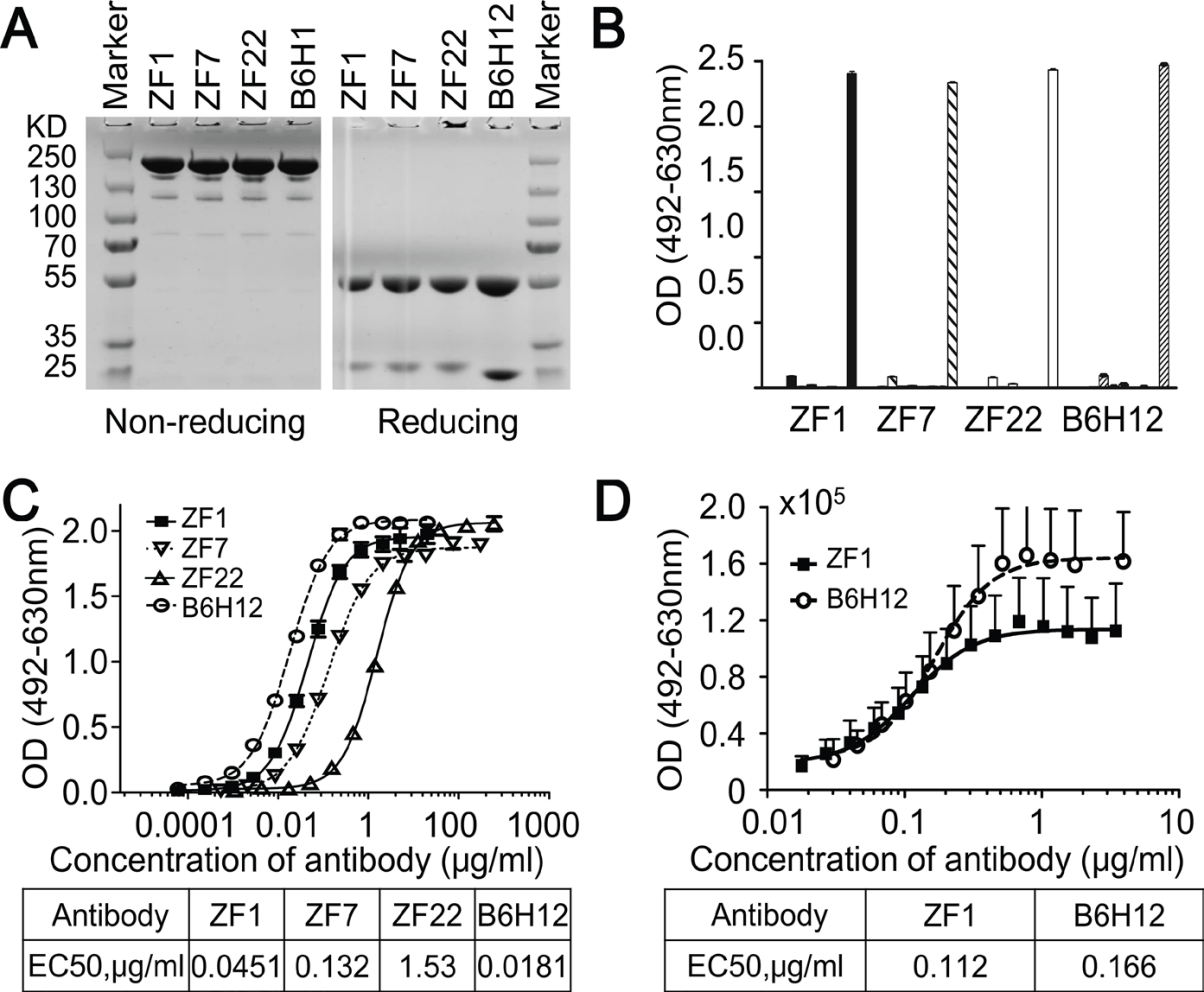
Isolation of human anti-CD47 antibodies (**A**) SDS-PAGE analysis results of the purified antibodies (IgG) under non-reducing and reducing conditions. (**B**) Isolated antibodies bound to recombinant CD47 specifically analyzed by direct ELISA. The antigens from left to right were PBS (as blank control), VEGF, KDR, IFN-γ, PDL1, HER2, Casein, BSA, and CD47 for each antibody. (**C**) Isolated antibodies bound to recombinant CD47 in a dose-dependent manner by direct ELISA. (**D**) ZF1 bound to cell-surface CD47 in a dose-dependent manner by Flow Cytometry. B6H12 served as positive control antibody in all assays.

To isolate high-affinity antibodies for CD47, Three sub-libraries including λ3-H3, λ3-H5 and λ1-H3 germline framework genes were screened with recombinant CD47 protein, and three rounds of panning were performed as previously described [36]. The titer of the eluted phages increased significantly over panning, indicating enrichment in CD47-specific binders. Hundreds of clones were analyzed by phage ELISA and the positive rate was 80%, from which twenty nine positive clones of unique genes were identified. These genes were then cloned into full-length human IgG1 expression vector and expressed in the FreeStyleTM 293-F system (Figure 1A). In addition, three well expressed IgG antibodies (named ZF1, ZF7 and ZF22) were identified as having specific binding activity to CD47 (Figure 1B).

### ZF1 specifically binds CD47 with high affinity

**Figure 2:**
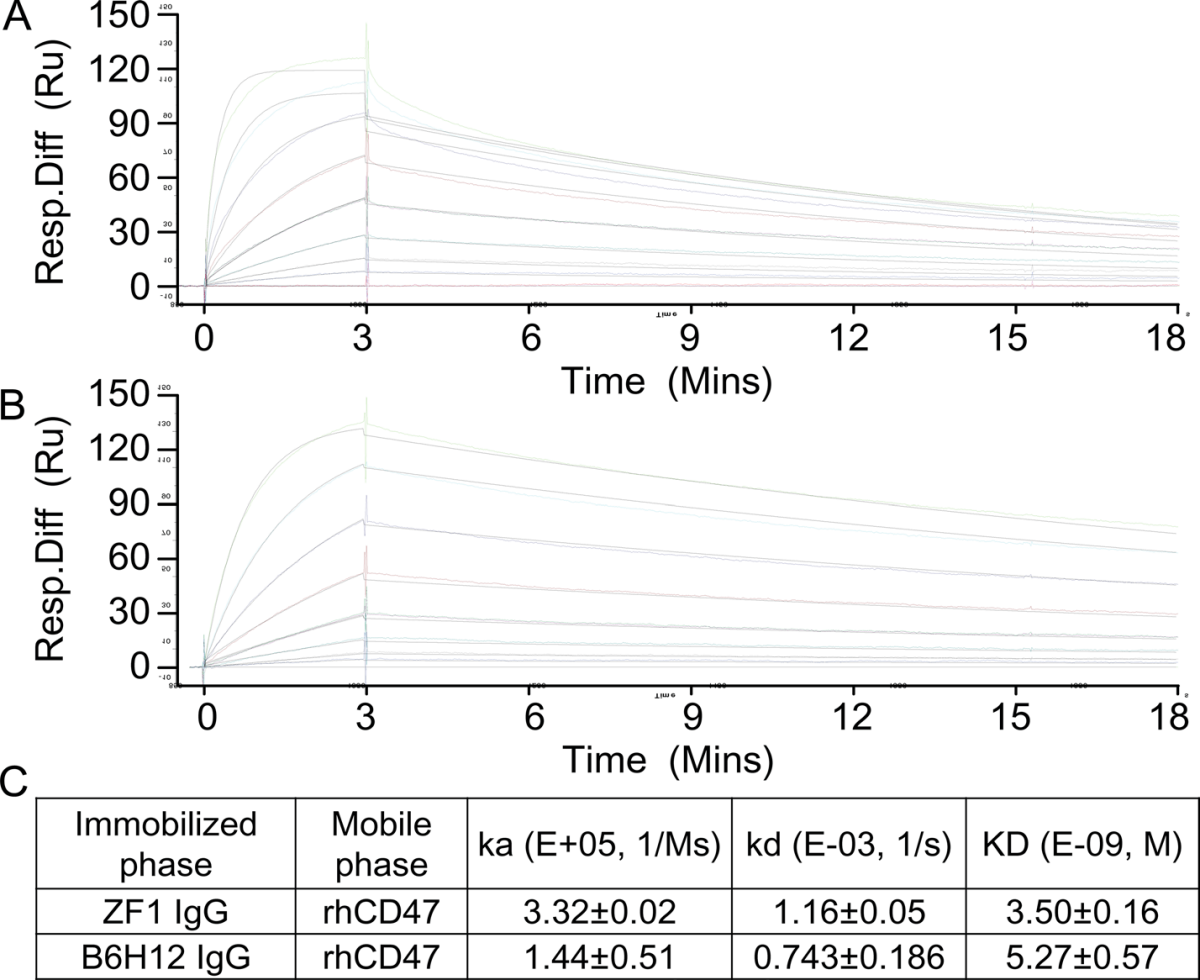
Affinity determination by Surface Plasmon Resonance (SPR) (**A** and **B**) Real time response curves of ZF1 and B6H12. Antibody concentrations were 200, 100, 50, 25, 12.5, 6.25 and 3.13 nM respectively. (**C**) Kinetic constants of ZF1 and B6H12 interacting with recombinant human CD47 extracellular region.

To examine the binding affinity between candidate antibodies and CD47, ELISA was first performed with recombinant CD47 protein. As shown in Figure 1C, ZF1, ZF7 and ZF22 all bound with the recombinant CD47 in a dose-dependent manner, while only ZF1 had an OD_50_ similar to B6H12, which is a humanized antibody targeting CD47 with impressive bioactivity *in vitro* and *in vivo* [5, 25]. Therefore, ZF1 was chosen for further analysis. Although flow cytometry analysis showed that the maximal binding of ZF1 to natural CD47 on cell surface was a bit weaker than the reported B6H12 antibody, there was no significant difference in EC50 between ZF1 (0.112) and B6H12 (0.166) (Figure 1D), indicating that there might be distinction between their binding mode to natural and recombinat CD47 protein. The affinity of ZF1 to CD47 was further determined by surface plasmon resonance (SPR) analysis using the BIAcore TM 3000 system. The kinetics constant of ZF1 with recombinant CD47 was 3.50 ± 0.16 nM, approaching that of B6H12 (5.27 ± 0.57 nM), with a faster on-rate as well as off-rate (Figure 2), and much higher than reported affinity of CD47 to SIRPα [27, 33].

### ZF1 treatment induces macrophage-mediated phagocytosis

**Figure 3:**
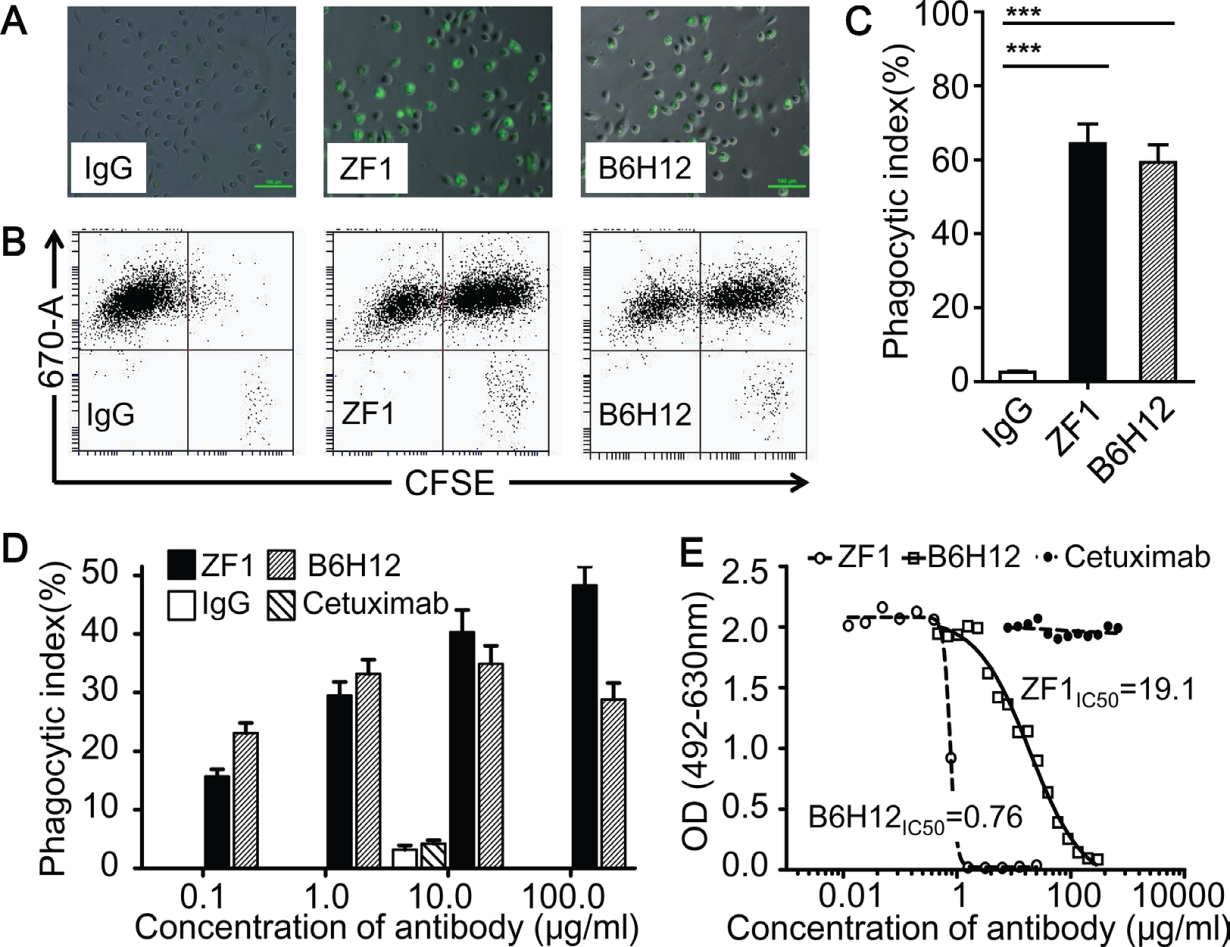
ZF1 induced antibody-dependent macrophage phagocytosis (**A**, **B** and **C**) Representative results for phagocytosis of CFSE-labeled CCRF cells phagocytosed by Dye eFluor^®^ 670-labeled macrophages. The results were firstly recorded by image (A), then analyzed by Flow Cytometry (B) and showed in a bar graph (C). (**D**) Anti-CD47 antibodies induced phagocytosis of U937 by macrophage at dose-dependent manner. Human IgG and anti-EGFR antibody Cetuximab were set as negative control at 10 μg/ml. (**E**) ZF1 blocked interaction between recombinant human CD47and recombinant human SIRPα.

We then examined whether ZF1 could functionally block the interaction between CD47 and SIRPα, which were known to inhibit macrophage-mediated phagocytosis of CD47^+^ cancer cells. As shown in Figure 3A–3D, ZF1 treatment induced efficient engulfment of CCRF and U937, two leukemic cells expressing high level of CD47 on cell surface. And the effects of phagocytosis were dose-dependent (Figure 3D). Consistent with robust phagocytosis induction, ZF1 antibody could efficiently block the physical interaction of immobilized recombinant human CD47 to human and mouse SIRPα in blocking assay *in vitro* (Figure 3E, Supplementary Figure S1). Interestingly, we found that although showing inferior blocking performance than B6H12 *in vitro* (Figure 3E, Supplementary Figure S1), ZF1 could induce macrophage-mediated phagocytosis as efficiently as did B6H12, or even more (Figure 3A–3C), which suggests that the biochemical assay may not always read out functional outcomes.

### Human AML and ALL xenograft models in BALB/c nude mice

**Figure 4:**
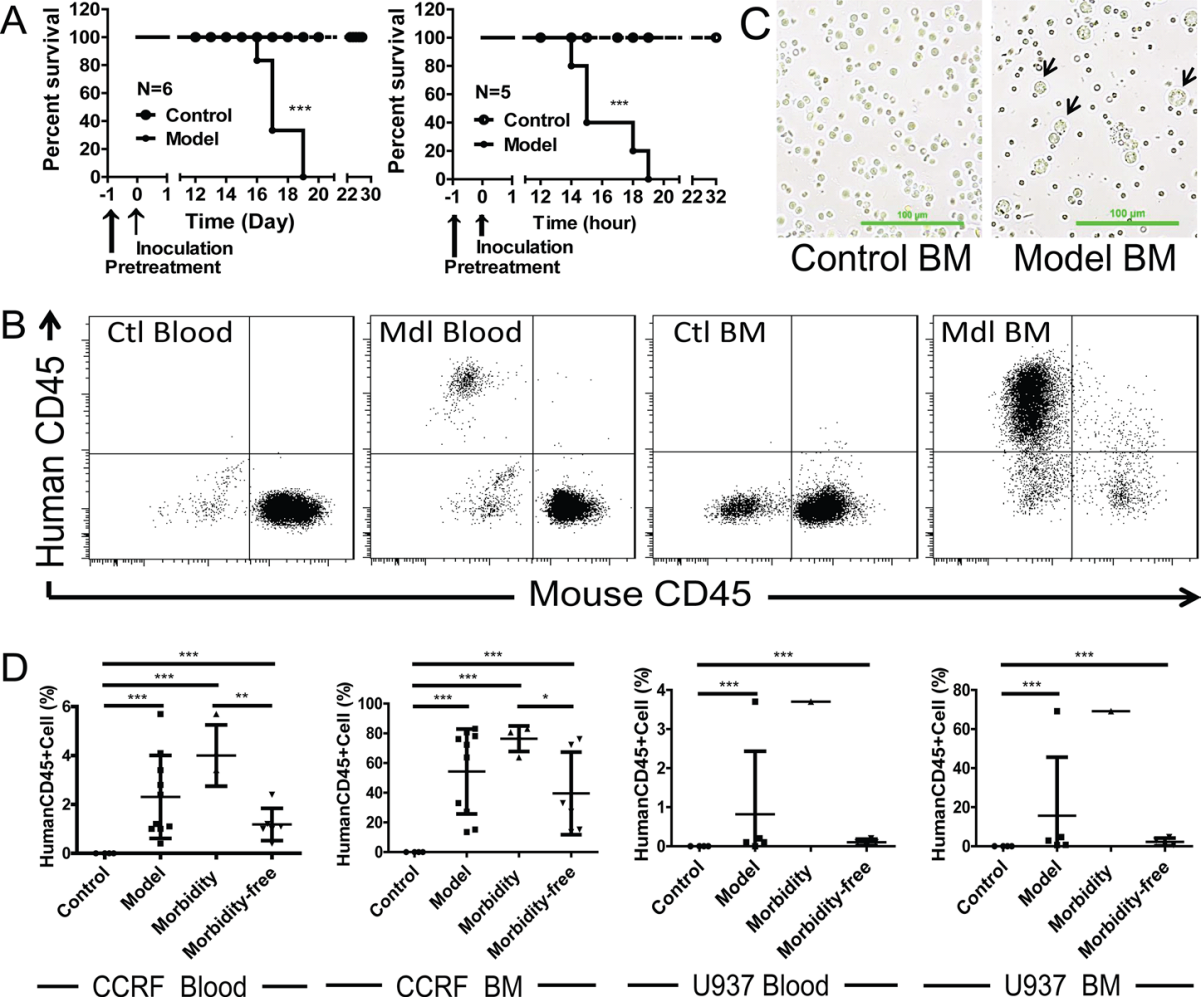
Engrafted mouse models for human ALL and AML (**A**) Kaplan-Meier plots of overall survival for BALB/c nude mice engrafted with CCRF cells for ALL (Left) and U937 cells for AML (Right). (**B**) Flow cytometry analysis of peripheral blood and bone marrow from CCRF-engrafted mouse model for the presence of leukemic cells (human CD45^+^). Data for U937 were not shown. (**C**) Representative images for bone marrow smear of U937-engrafted mouse model. U937 cells are bigger in size than the normal bone marrow cells. (**D**) Correlation of morbidity with the presence of leukemic cells in peripheral blood and bone marrow of CCRF and U937-engrafted mouse models. Ctl: control; Mdl: model; BM: bone marrow.

To investigate the anti-tumor activities of ZF1 *in vivo*, we first managed to establish ALL and AML xenograft models by using cell lines CCRF and U937 in BALB/c nude mice. As shown in Figure 4, intravenous injection of 1 **×** 10^7^ CCRF or U937 cells led to leukemia, characterized by population of CCRF or U937 cells in both peripheral blood and bone marrow (Figure 4B and 4C), and the death of all BALB/c nude mice within 3 weeks (Figure 4A). Additionally, according to morbidity epidemic level, model mice were divided into two subgroups (morbidity subgroup and morbidity-free subgroup) and morbidity was tightly correlated with the level of leukemic cells in both peripheral blood and bone marrow samples (Figure 4D). The results showed that the leukemia models were established successfully.

### Anti-Leukemia activity of ZF1 antibody

**Figure 5:**
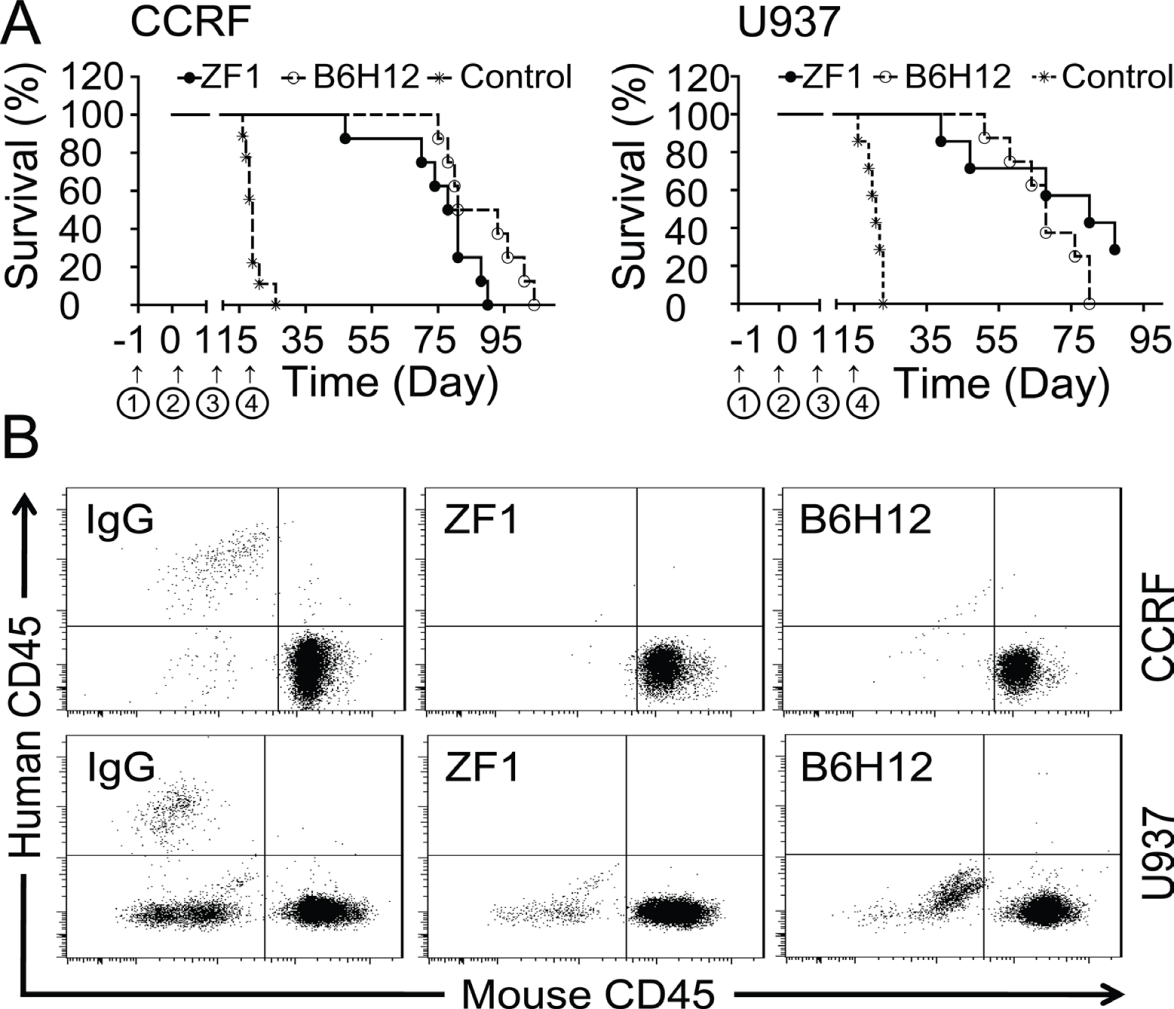
Protection from leukemic death by ZF1 ***in vivo*** (**A**) Kaplan-Meier plots of overall survival for BALB/c nude mice engrafted with CCRF cells for ALL (Left) and U937 cells for AML (Right) in the presence of anti-CD47 antibodies (ZF1 and B6H12). ① Pretreatment with cyclophosphamide. ② Inoculation of Leukemia cell. ③ Start administration. ④ Stop administration. (**B**). Flow cytometry analysis of peripheral blood from leukemic cells-engrafted mouse models for the presence of leukemic cells (human CD45^+^) after anti-CD47 antibody treatment.

Utilizing the models established above, we tested the protective effects of ZF1 antibody. Mice of the control group administered with IgG died successively in the third or fourth week after inoculation, while the mice administered with ZF1 were still surviving and healthy after six weeks (Figure 5A), in agreement with which, no leukemic cells were detected in the blood of treatment groups four weeks after cells were inoculated (Figure 5B) while could be readily detected in IgG-treated mice (Figure 5B). However, both ZF1 and B6H12 could not cure the mice completely and the mice died for leukemia progress eventually under the current conditions. Despite death occurring first in the ZF1 group, there was no significant difference for ZF1 and B6H12 in protecting mice from leukemia death (Figure 5A).

**Figure 6:**
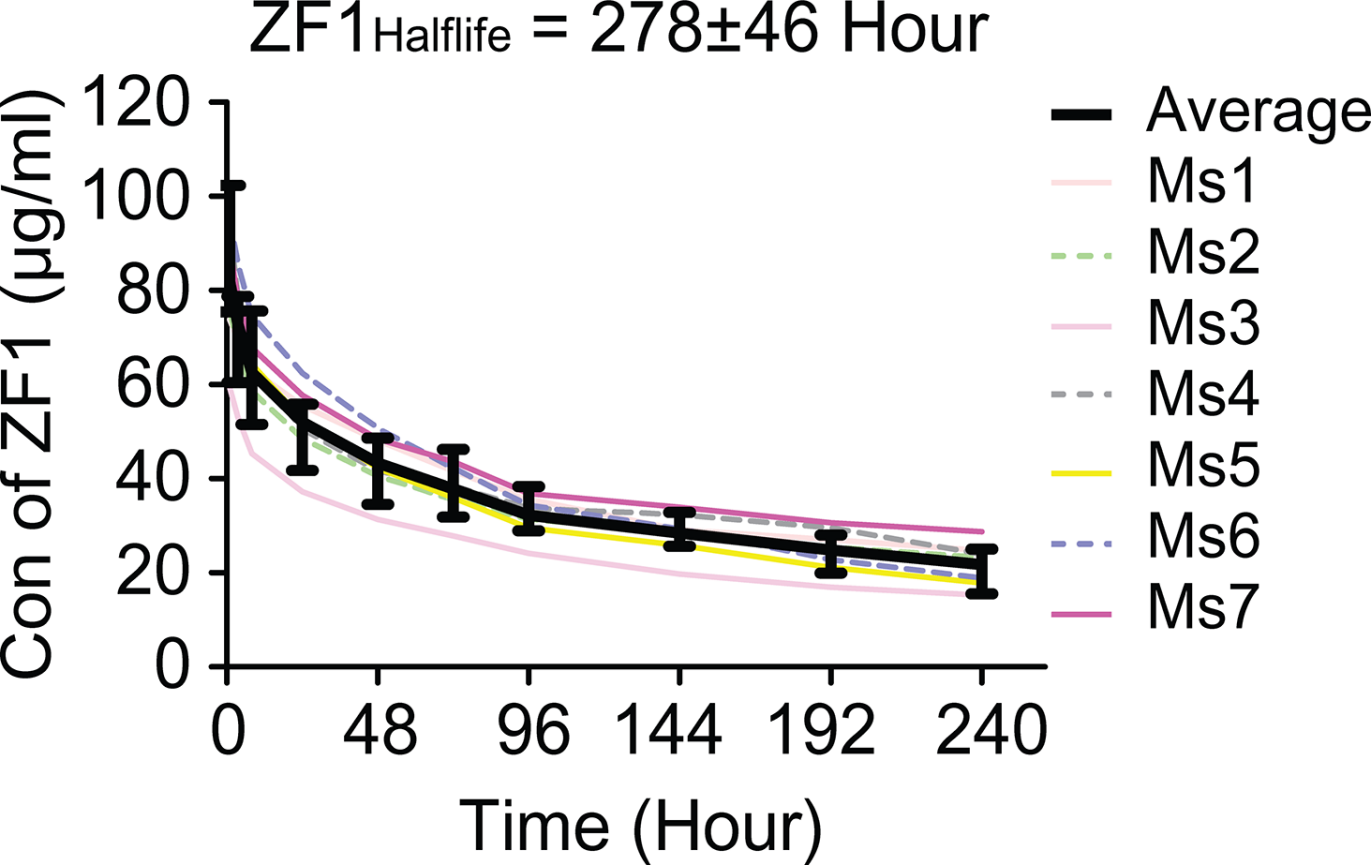
Pharmacokinetic analysis of ZF1 The average half-life of ZF1 is 275 ± 60 hours. Colored curves are for individual mouse (Ms) while black curve with SD for the averaged value (Ave).

The pharmacokinetics of ZF1 was investigated by intravenous injection of single dose of ZF1 (10 mg/kg, *n* = 7) into BALB/c mice. The half-life of ZF1 was determined to be 275 ± 60 hours (Figure 6), which was long enough for bio-activation evaluation *in vivo*. ZF1 concentration was also examined 24 hours after the third injection in the xenograft tumor assay. All the samples contained ZF1 at concentration of 95.7 ± 12.2 μg/ml, which was above the reported effective concentration of 50 μg/ml [34], while B6H12 was 125.7 ± 22.2 μg/ml. These results underlie the anti-leukemia activity of ZF1.

## DISCUSSION

CD47 is one of the critical molecules in immune evasion. Cancer cells could escape phagocytosis via CD47 overexpression, which in turn signaling to macrophage via binding SIRPα on cell surface [9–11, 26]. Antibodies or other blocking agents targeting CD47/SIRPα could block communication between cancer cells and macrophages and induce phagocytosis, potentially an effective method of cancer therapy [10, 11, 19, 25, 27]. The purpose of this study was to generate human anti-CD47 antibodies and determine their biological effects. To our knowledge, ZF1 is the first human anti-CD47 antibody isolated from phage display library with biological activities both *in vitro* and *in vivo*.

The CD47 protein contains three domains. One of which, the IgV-like extracellular domain, has been previously demonstrated to bind directly with SIRPα [2, 37]. Therefore, the IgV-like extracellular domain was used for antibody screening and ZF1 was selected for its high affinity and specificity. It was also previously reported that antibody could promote phagocytosis by blocking CD47/SIRPα interaction [19, 25]. Our research demonstrated that ZF1 could block interaction between human CD47 and human (or mouse) SIRPa, although a bit weaker than B6H12 in biochemical assay (Figure 3E and Supplementary Figure S1), but induce macrophage-mediated phagocytosis as robust as B6H12 did (Figure 3A–3C). For the surprising outcomes, it is conceivable that the recombinant CD47 used in blocking assays may not be structurally identical to the natural CD47 on cancer cell surface considering differences in modification. Another factor potentially accountable for this obvious discrepancy is binding epitopes. However, the competitive binding assay do not support this idea well as ZF1 could inhibit FTTC conjected B6H12 binding to natural CD47 on cell surface (Supplementary Figure S2) and pre-incubation of recombinant CD47 with B6H12 antibody prevented it from further binding with ZF1 (Supplementary Figure S3), suggesting that B6H12 and ZF1 may actually, or at least partially, bind a similar epitope on CD47. Currently, we could not rule out the possibility that recombinant CD47 may not be the best surrogate for natural CD47 on cancer cell surface. Nevertheless, we did find that ZF1 could induce phagocytosis and kill leukemic cells as effectively as B6H12 antibody. In addition, considering the ceiling average fluorescence intensity of ZF1 is lower than B6H12 (Figure 1D) irrespective of the high concentrations, we also hypothesized that rapid endocytosis of ZF1/CD47 complex might be a potential underlying mechanism according to previous reports [38, 39]. Consistent with this idea, our preliminary data showed ZF1 seemed to be more effective than B6H12 to promote the internalization of CD47 (Supplementary Figure S4). And more experimental evidence is warrant to address this issue.

Additionally, fine pharmacokinetics is a key character for candidate drug potency. Our preliminary pharmacokinetics assay result showed that ZF1 has a half-life of 275 ± 60 hours (Figure 6), which is long enough for *in vivo* bioactivity evaluation in mice. As ZF1 cannot binding to mouse CD47 (Supplementary Figure S5), the ligand introduced antibody consumption could not be accessed here and the half-life could not reflect the actual situation in human. Pharmacokinetics assays in primates, of which the CD47 is more homologous to human CD47, would be more suitable for estimating the accurate half-life.

Recently, blocking CD47 was found resulting in T cell activation [28, 29]. In this work, ZF1 showed potent anti-leukemia activities in nude mice, but its effects on T cell activation could not be examined in these models. Nevertheless, we hypothesize ZF1 might display stronger anti-tumor effects when T cells were activated by tumor-antigen presentation induced by the enhanced phagocytosis. Such experiments are in consideration for the future.

Interestingly, Macrophages were recently reported involving in cell-in-cell structures in solid tumors [40, 41]. Cell-in-cell structures, characterized by one or more viable cells present inside another cell, were frequently formed between tumor cells and usually led to the death of inner cells [42]. Latest researches indicated that cell-in-cell formation by entosis is a key mechanism of cell competition to promote clonal selection and tumor evolution [42–44]. Despite being reported over a century, cell-in-cell remains largely mysterious in its forming mechanisms although progress were made recently [45–47]. Since blocking CD47 by antibodies could efficiently induce macrophage-mediated phagocytosis of tumor cells and treat cancers, it would be interesting to examine whether CD47 also participate in cell-in-cell formation between tumors, and if so, would blocking CD47 a feasible way to inhibit tumor growth by inducing cell-in-cell formation and the mediated-cell death?

## MATERIALS AND METHODS

### Materials

Human antibody library with a high-capacity of 1.35 × 10^10^ was constructed by Beijing bio-engineering institute (ZL200910091261.8). Recombinant human CD47 and SIRPα, both fused with His tag or human Fc, were obtained from ACRO biosystems. Helper phage M13KO7 was from Invitrogen. CCRF, U937 and SKOV-3 were kind gifts from Dr. Zhixin-Yang and professor Qinong-Ye, respectively (Beijing bio-engineering institute). All cell lines were maintained in 1640 medium supplemented with 10% FBS (Fetal Bovine Serum; Gibco) at 37°C and 5% CO_2_ as described in ATCC. HEK293T cells used for eukaryotic protein expression were from Invitrogen and maintained in FBS free medium at 37°C and 5% CO_2_. Eukaryotic cell expression vectors were constructed and stored in our laboratory. Fluorescent substance labeled flow cytometry antibodies were products of eBiosciences.

All the mice in this study were purchased from the laboratory animal center, Academy of military medical sciences or bred in house. All mouse experiments were conducted according to an Institutional Animal Care and Use Committee–approved protocol. Animals were euthanized per institutional protocols.

### Selection of phage human antibody library against CD47

Phage human antibody selection was performed as previously described [36]. Three rounds of selection were executed in immune tubes (Nunc, 443990) coated with recombinant CD47-his (ACRO Biosystems, CD7-H5227) using the phage displayed single-chain variable fragment (scFv) library. Next, individual XL-Blue clones recovered from the third round of selection were randomly picked and identified for binding ability and specificity by phage ELISA. DNA sequences of positive monoclonal scFv were determined by dideoxynucleotide sequencing and analysis of the antibody genes were performed using DNA Club.

### Expression and purification of the full-length IgG

Genes encoding the heavy (VH) and light (VL) chain were amplified by primers pairs and cloned into a pair of eukaryotic cell expression vectors. The recombinant light and heavy chain containing vectors were co-transfected into FreeStyle HEK293T cells (Invitrogen, K1548) for instantaneous expression. Expression supernatants, containing different IgG proteins, were collected by centrifugation and purified using HiTrap protein A FF 1-mL column (GE Healthcare). Purified antibodies were identified by 10% non- reduced and DTT-reduced SDS-PAGE.

### CD47 binding and blocking activity assay by ELISA

In the direct binding assay, 96-well Maxisorp microtiter plates were pre-coated with CD47 (100 ng in 50 μL PBS per well) and pre-blocked with 2% milk in PBS solution. Then, various amounts of anti-CD47 antibodies were added and incubated at 37°C for 1 hour. Subsequently the plates were washed 5 times with PBST and incubated with 100 μl of a HRP conjugated goat anti-human Fc secondary antibody (ZSGB-BIO) at 37°C for 30 minutes. The remaining ELISA steps were completed following the procedures previously described for the phage ELISA. OD50, the antibody concentration required for half of max absorbance, was then calculated by GraphPad Software.

In the competitive CD47/SIRPα blocking assay, various amounts of anti-CD47 antibodies were mixed with a fixed amount of CD47-Fc (100 ng/ml) and incubated at 37°C for 1 hour. The mixture was then transferred to 96-well microtiter plates pre-coated with SIRPα (200 ng/well) and incubated at 37°C for 1 hour. Next, the plates were washed preceding addition of the secondary antibody. Subsequently, activity was detected via OPD chromogenic reaction as previously described, and IC50, the antibody concentration required for 50% inhibition of Fc-CD47/SIRPα reaction, was calculated by GraphPad Software.

### Affinity determination via SPR

The affinity of antibodies was determined via SPR on a Biacore TM 3000 system. IgG capture antibody in the standard IgG capture antibody kit was immobilized on a CM5 chip using standard amino coupling kit. Anti-CD47 antibody was captured at a certain level (300 Ru) and reacted with recombinant CD47 at gradient concentrations (starting with 200 nM sequentially diluted to 3.1 nM) in fluid HBSEP buffer (PH 7.4). At the end of each circle, the captured antibody, along with CD47, was washed away with regeneration buffer and the chip was used for the next circle reaction until the test was completed. Then, the affinity was calculated in a 1:1(Langmuir) binding fit model by BIAevaluation Software.

### Cell ELISA and flow cytometry assay

Cell ELISA and flow cytometry assays were carried out for identification of the binding activity between the antibody and native CD47 on the cell surface. In cell ELISA, SKOV-3 cells were planted in 96-well plates at 5 × 10^3^ cells per well. The remaining ELISA steps followed the ELISA procedure described above except the test temperature was adjusted to 4°C to avoid endocytosis induced by CD47 on the cell surface. In flow cytometry, 5 × 10^5^ cells were incubated with anti-CD47 antibody sample in 100 μL at 4°C for 1 hour. Cells were then washed using flow cytometry staining buffer and incubated with a FITC labeled goat anti-human IgG secondary antibody in 100 μL. Lastly, the cells were washed and analyzed by flow cytometry.

### Blood and bone marrow flow cytometry

Peripheral blood samples of 20 μL were collected and mixed with 20 μL anti coagulation containing 1.8 mg/mL K2EDTA. Then, APC labeled anti-human CD45 antibody and PE labeled anti-mouse CD45 antibody were added in the blood samples and incubated for 30 minutes at room temperature. Finally, 2 mL lysis buffer was added in the samples and incubated for 10 minutes at room temperature before analysis by flow cytometry. Identically, bone marrow samples were collected and suspended in flow cytometry staining buffer. The samples were processed and analyzed following the procedures used for blood samples.

### Phagocytosis assay *in vitro*

For phagocytosis assay *in vitro*, similarly to previously described [25], macrophages isolated from mice abdominal cavity were labeled with 1 μM Cell Proliferation Dye eFluor^®^ 670 according to the manufacturer's protocol (eBioscience). Macrophages were plated at 1×10^5^ per well in a 6-well tissue-culture plate overnight and incubated in serum-free medium for 2 hours before adding tumor cells. Tumor cells were labeled with 1 μM 5-(and 6)-Carboxyfluorescein diacetate succinimidyl ester (CFSE, eBioscience), and incubated with anti-CD47 antibodies for 1 hour at 37°C. Subsequently, tumor cells were added into macrophages planted plates and incubated 2–4 hours. Next, macrophages were repeatedly washed and imaged with an inverted fluorescence microscope (Leica MI6000B). Lastly, macrophages were digested and analyzed by flow cytometry. The phagocytic index was calculated as the number of phagocytosed tumor cells (CFSE^+^ cells) per 100 macrophages (670-A^+^ cells).

### Pharmacokinetics assay

The PK of ZF1 was evaluated in a similar method as previously described [48]. 10 mg/kg ZF1 was administered by tail vein injection into BALB/c mice (*n* = 3). Blood samples were collected via the caudal vena cava at 15 minutes, 1 hour, 3.5 hours, 8 hours, 1day, 2 days, 3 days, 4 days, 5 days, 7 days, and 9 days after injection. Blood samples were incubated at room temperature for 30 min and centrifuged to collect supernatant. To measure the serum concentrations, direct binding assay was carried out as described. The serum samples were diluted appropriately and a calibration curve was used to calculate the concentrations. The calibration curve points were 15.6, 31.3, 62.5, 125, 250, 500, 1000 and 2,000 ng/mL (15.6 and 2000 ng/mL as anchor points). Serum concentrations of ZF1 were interpolated from a 4-parameter logistic fit of the standard curve on the same plate and non-compartmental PK parameters were calculated.

### Anti-leukemia activity assay *in vivo*

Female BALB/c nude mice, 6~8 weeks old, were used for the anti-leukemia activity assay. Firstly, cyclophosphamide was administrated with a single dose of 3 mg per mouse via intraperitoneal injection. Twenty four hours later, mice were inoculated intravenously with 1 × 10^7^ALL CCRF cells or AML U937 cells. One day after cell inoculation, mice of therapy groups were treated daily with 100 μg (per mouse) of anti-CD47 antibodies via intraperitoneal injection for 14 days, while mice of the control group were treated with human IgG. Mice were observed daily for morbidity and time of survival was recorded. Additionally, an independent group of model mice were set up. When morbidity was observed, the mice were euthanized and dissected for pathology analysis.

### Statistics

Statistical comparison of phagocytosis index and proportion of leukemia cell in blood or marrow samples were analyzed by one-way analysis of variance (ANOVA). Mutiple measurements date was analyzed by Wilcoxon signed rank test. Over survival date was analyzed by the Kaplan-Meier survival estimates and the statistical significance of differences in overall survival was calculated by the Mantel-Cox log-rank test. A significance level of *p* < 0.05 was used for all tests. GraghPad Prism 5 statistic software was used for all statistical analyses.

## SUPPLEMENTARY MATERIALS


