# Supplementary material for: A fully human anti-CD47 blocking antibody with therapeutic potential for cancer
Source: Oncotarget. 2016 Nov 15;7(50):83040–50. doi: 10.18632/oncotarget.13349 (PMC5347751; doi:10.18632/oncotarget.13349)
Supplement: Supplementary file 1 [file oncotarget-07-83040-s001.pdf]

A fully human anti-CD47 blocking antibody with therapeutic potential for cancer

Supplementary Materials

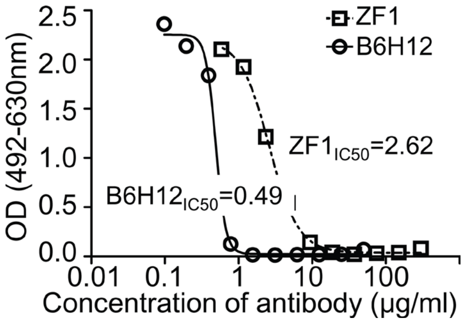

Supplementary Figure S1: ZF1 blocked interaction of recombinant human CD47 and recombinant mice SIRPα. The IC50 was 2.62 μg/ml for ZF1 vs 0.49 μg/ml for B6H12, while it was 19.1 μg/ml for ZF1 vs 0.76 μg/ml for B6H12 in Figure 3E for blocking human CD47 and recombinant human SIRPα.

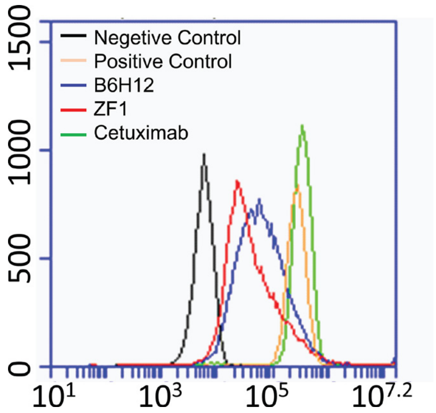

Supplementary Figure S2: ZF1 inhibited FTTC conjected B6H12 binding to natural CD47 on cell surface. U937 cells were firstly incubated with ZF1, B6H12 or Cetuximab (Cetuximab acts as negative control) at working concentration of 500 μg/ml at 4°C for 1 hour, and then incubated with subsequently added FTTC conjected B6H12 at 10 μg/ml for 30 mins. Finally the binding intensity of FTTC conjected B6H12 to U937 cells was detected by flow cytometry assay. The results showed that both ZF1 and B6H12 could inhibit FTTC conjected B6H12 from binding to U937. The inhibition activity of ZF1 was even more effectively than B6H12 self.

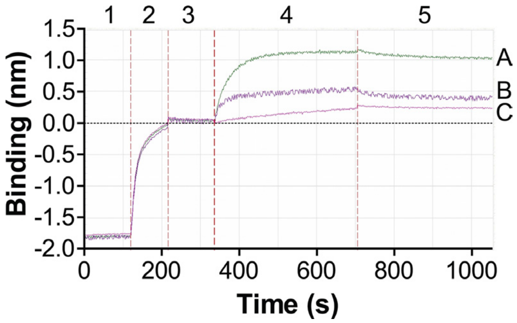

Supplementary Figure S3: B6H12 inhibits recombinat human CD47 binding with ZF1. The competitive binding assay was carried out via Biolayer Interferometry (BLI) technique using the equipment of Fortebio Octet QKe. 4 μM Fab of B6H12 or ZF1 was mixed with 100 nM recombinat human CD47 (rhCD47) and incubated for 30 mins at 37℃, then the mixture samples reacted with IgG of ZF1 which was captured on standardized sensors pre-coated with anti-human Fc antibody. 4 μM Fab of cetuximab mixed with 100 nM rhCD47 acted as negative control. Real-time reaction data was recorded and output as a time-response curve. The arabic numerals upward show the assay steps. Anti-human capture sensors were preprocessed in HBSEP buffer in Step 1 and then IgG of ZF1 was captured in step 2. Subsequently, a baseline process was carried out in step 3 and Fab/rhCD47 mixture samples reacted with ZF1 captured on sensor in step4. Finally a dissociation process was carried out in step 5. Curve A is for cetuximab Fab, Curve B for ZF1 Fab and Curve C for B6H12 Fab. The results showed that B6H12, as well as ZF1 self, was able to block the interaction of ZF1/ rhCD47.

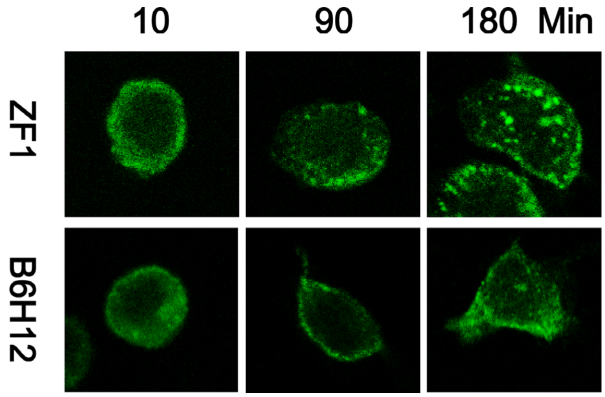

Supplementary Figure S4: ZF1 enhanced cell-surface-CD47 endocytosis *in vitro*. SKOV-3 cells were planted in chamber slide (5000 cells/well) and cultivated overnight. Then the cells were starved for 2 hours in medium free of FBS and subsequently co-incubated with 20 μg/ml FITC-labeled ZF1 or B6H12 for 10, 30, 60, 90, 120 and 180 mins. Finally cells were fixed on the slide, observed and photographed using confocal microscope. After 180 mins' co-incubation, distinctly green fluorescence plots could be observed intra cells in group ZF1 but barely observed in group B6H12.

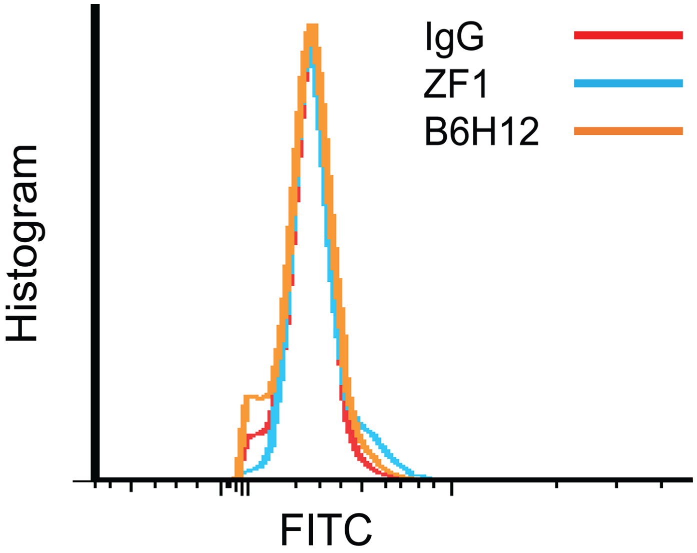

Supplementary Figure S5: Both ZF1 and B6H12 could not bind with mouse CD47. Mouse red cells were incubated with ZF1, B6H12 or human IgG at 10 μg/ml at 4°C for 1 hour, and then cells were washed and subsequently incubated with FTTC conjected goat anti-human IgG antibody for 30 mins. Finally the mouse red cells were detected via flow cytometry assay. The result showed that both ZF1 and B6H12 could not bind with mouse CD47 on red cell surface.
